# Supplementary material for: Simulation of Closed-Loop Deep Brain Stimulation Control Schemes for Suppression of Pathological Beta Oscillations in Parkinson’s Disease
Source: Front Neurosci. 2020 Mar 5;14:166. doi: 10.3389/fnins.2020.00166 (PMC7066305; doi:10.3389/fnins.2020.00166)
Supplement: Supplementary file 1 [file Data_Sheet_1.PDF]

Supplementary Material

1 Supplementary Data

The transmembrane potential of each neuron (v_m) from each population in the network model is described by a Hodgkin-Huxley type neuron model. Details of each neuron model are described below.

1.1 STN Neuron Model

The STN model used is from (Otsuka et al., 2004) using the implementation from Hahn and McIntyre (2011). The membrane potential of an STN neuron is described by

$$C_m \frac{dv_m}{dt} = -I_l - I_{Na} - I_K - I_A - I_L - I_T - I_{Ca-K} - \sum_k I_{syn}^k + I_{app} \qquad (A.1)$$

where C_m is the membrane capacitance, I_l is the leak current, I_{Na} is a sodium current, I_K is a Kv3-type potassium current, I_A is a voltage dependent A-type potassium current, I_L is an L-type long lasting calcium current, I_{Ca-K} is a calcium activated potassium current, I_{syn} are synaptic currents and I_{app} is the neuron bias current. The equations governing the ionic currents are detailed in Table 1.

Table 1: STN Neuron Model Equations

Current	Equation
$\boldsymbol{I_l}$	$g_l(v_m - E_l)$
$\boldsymbol{I_{Na}}$	$g_{Na}m^3h(v_m - E_{Na})$
$\boldsymbol{I_K}$	$g_K n^4(v_m - E_K)$
$\boldsymbol{I_A}$	$g_A a^2 b(v_m - E_K)$
$\boldsymbol{I_L}$	$g_L c^2 d_1 d_2(v_m - E_{Ca})$
$\boldsymbol{I_T}$	$g_T p^2 q(v_m - E_{Ca})$
$\boldsymbol{I_{Ca-K}}$	$g_{Ca-K} r^2(v_m - E_K)$

The gating kinetics of the ionic conductances are governed by

$$\frac{dx}{dt} = \frac{x_\infty - x}{\tau_x} \qquad (A.2)$$

where x stands for a, b, c, d_1, d_2, h, m, n, p, q, or r. The steady state activation and inactivation functions are given by

$$x_\infty = \frac{1}{1 + e^{\frac{v+\theta_x}{k_x}}} \qquad (A.3)$$

where θ_x and k_x are the half-activation/half-inactivation voltage and slopes, respectively. The activation time constants are given by

$$\tau_x = \tau_x^0 + \frac{\tau_x^1}{e^{-\frac{(v+\theta_x^1)}{\sigma_x^1}} + e^{-\frac{(v+\theta_x^2)}{\sigma_x^2}}} \quad (A.4)$$

The dynamics of the intrinsic calcium level $[Ca]_i$ are described by

$$\frac{d[Ca]_i}{dt} = \epsilon_{Ca}\left(-\frac{I_T + I_L}{2F} - k_{Ca}[Ca]_i\right) \quad (A.5)$$

where $[Ca]_i$ is the intrinsic calcium concentration, ϵ_{Ca} is a constant which accounts for the combined effects of intracellular calcium buffering mechanisms and cellular geometry, F is Faraday's constant and k_{Ca} is the calcium pump rate. Parameter values for the STN neuron model are summarized in Table 2.

Table 2: STN Neuron Model Parameters

Parameter	Values	Parameter	Values	Parameter	Values
$\boldsymbol{C_m}$	1 µF/cm^2	θ_r	0.17e-3 mM	τ_r	2 ms
$\boldsymbol{L}$	60 µm	k_a	-14.7 mV	θ_a^τ	-40 mV
$\boldsymbol{Diam}$	60 µm	k_b	7.5 mV	$\theta_b^{\tau1}, \theta_b^{\tau2}$	-60, -40 mV
$\boldsymbol{g_l}$	0.35 mS/cm^2	k_c	-5 mV	$\theta_c^{\tau1}, \theta_c^{\tau2}$	-27, 50 mV
$\boldsymbol{g_{Na}}$	49 mS/cm^2	k_{d_1}	7.5 mV	$\theta_{d_1}^{\tau1}, \theta_{d_1}^{\tau2}$	-40, -20 mV
$\boldsymbol{g_K}$	57 mS/cm^2	k_{d_2}	0.02 µM	$\theta_h^{\tau1}, \theta_h^{\tau2}$	-50, -50 mV
$\boldsymbol{g_A}$	5 mS/cm^2	k_h	6.4 mV	θ_m^τ	-53 mV
$\boldsymbol{g_L}$	15 mS/cm^2	k_m	-8 mV	$\theta_n^{\tau1}, \theta_n^{\tau2}$	-40, -40 mV
$\boldsymbol{g_T}$	5 mS/cm^2	k_n	-14 mV	$\theta_p^{\tau1}, \theta_p^{\tau2}$	-27, -102 mV
$\boldsymbol{g_{Ca-K}}$	1 mS/cm^2	k_p	-6.7 mV	$\theta_q^{\tau1}, \theta_q^{\tau2}$	-50, -50 mV
$\boldsymbol{E_l}$	-60 mV	k_q	5.8 mV	σ_a	-0.5 mV
$\boldsymbol{\theta_a}$	-45 mV	k_r	-0.08 µM	σ_b^1, σ_b^2	-30, 10 mV
$\boldsymbol{\theta_b}$	-90 mV	τ_a^0, τ_a^1	1, 1 ms	σ_c^1, σ_c^2	-20, 15 mV
$\boldsymbol{\theta_c}$	-30.6 mV	τ_b^0, τ_b^1	0, 200 ms	$\sigma_{d_1}^1, \sigma_{d_1}^2$	-15, 20 mV
$\boldsymbol{\theta_{d_1}}$	-60 mV	τ_c^0, τ_c^1	45, 10 ms	σ_h^1, σ_h^2	-15, 16 mV
$\boldsymbol{\theta_{d_2}}$	0.1 µM	$\tau_{d_1}^0, \tau_{d_1}^1$	400, 500 ms	σ_m	-0.7 mV
$\boldsymbol{\theta_h}$	-45.5 mV	τ_h^0, τ_h^1	0, 24.5 ms	σ_n^1, σ_n^2	-40, 50 mV

$\boldsymbol{\theta_m}$	-40 mV	τ_m^0, τ_m^1	0.2, 3 ms	σ_p^1, σ_p^2	-10, 15 mV
$\boldsymbol{\theta_n}$	-41 mV	τ_n^0, τ_n^1	0, 11 ms	σ_q^1, σ_q^2	-15, 16 mV
$\boldsymbol{\theta_p}$	-56 mV	τ_p^0, τ_p^1	5, 0.33 ms	I_{app}	-0.125 nA
$\boldsymbol{\theta_q}$	-85 mV	τ_q^0, τ_q^1	0, 400 ms		

where *L* and *Diam* represent the length and diameter of the single compartment STN neuron.

1.2 Globus Pallidus Neuron Model

The GPe and GPi models used are from (Rubin and Terman, 2004; Terman et al., 2002) using the implementation from Hahn and McIntyre (2011). The membrane potential of an GP neuron is described by

$$C_m \frac{dv_m}{dt} = -I_l - I_{Na} - I_K - I_T - I_{Ca} - I_{AHP} - \sum_k I_{syn}^k + I_{app} \qquad (A.6)$$

where C_m is the membrane capacitance, I_l is the leak current, I_{Na} is a sodium current, I_K is a potassium current, I_T is a low-threshold T-type calcium current, I_{Ca} is a high-threshold calcium current, I_{AHP} is a calcium activated, voltage-independent 'afterhyperpolarization' potassium current, I_{syn} are synaptic currents and I_{app} is the neuron bias current. The equations governing the ionic currents are detailed in Table 1.

Table 3: GP Neuron Model Equations

Current	Equation
$\boldsymbol{I_l}$	$g_l(v_m - E_l)$
$\boldsymbol{I_{Na}}$	$g_{Na}m^3h(v_m - E_{Na})$
$\boldsymbol{I_K}$	$g_K n^4(v_m - E_K)$
$\boldsymbol{I_T}$	$g_T p^2 q(v_m - E_{Ca})$
$\boldsymbol{I_{AHP}}$	$g_{AHP} r^2(v_m - E_K)$

The gating kinetics of the ionic conductances are governed by

$$\frac{dx}{dt} = \frac{x_\infty - x}{\tau_x}$$

where *x* stands for *h*, *m*, *n*, *p*, *q*, or *r*. The steady state activation and inactivation functions are given by

$$x_\infty = \frac{1}{1 + e^{(v+\theta_x)/k_x}}$$

where θ_x and k_x are the half-activation/half-inactivation voltage and slopes, respectively. The activation time constants are given by

$$\tau_x = \tau_x^0 + \frac{\tau_x^1}{e^{-\frac{(v+\theta_x^1)}{\sigma_x^1}} + e^{-\frac{(v+\theta_x^2)}{\sigma_x^2}}} \qquad (A.7)$$

Parameter values for the GP neuron model are summarized in Table 4:

Table 4: GP Neuron Model Parameters

Parameter	Values	Parameter	Values	Parameter	Values
$\boldsymbol{C_m}$	1 µF/cm^2	θ_q	-85 mV	τ_r	2 ms
$\boldsymbol{L}$	60 µm	θ_r	0.17 µM	$\theta_h^{\tau1}, \theta_h^{\tau2}$	-50, -50 mV
$\boldsymbol{Diam}$	60 µm	k_h	6.4 mV	θ_m^{τ}	-53 mV
$\boldsymbol{g_l}$	0.35 mS/cm^2	k_m	-7 mV	$\theta_n^{\tau1}, \theta_n^{\tau2}$	-40, -40 mV
$\boldsymbol{g_{Na}}$	49 mS/cm^2	k_n	-14 mV	$\theta_p^{\tau1}, \theta_p^{\tau2}$	-27, -102 mV
$\boldsymbol{g_K}$	57 mS/cm^2	k_p	-6.7 mV	$\theta_q^{\tau1}, \theta_q^{\tau2}$	-50, -50 mV
$\boldsymbol{g_T}$	5 mS/cm^2	k_q	5.8 mV	σ_h^1, σ_h^2	-15, 16 mV
$\boldsymbol{g_{AHP}}$	1 mS/cm^2	k_r	-0.08 µM	σ_m	-0.7 mV
$\boldsymbol{E_l}$	-60 mV	τ_h^0, τ_h^1	0, 4.5 ms	σ_n^1, σ_n^2	-40, 50 mV
$\boldsymbol{\theta_h}$	-45.5 mV	τ_m^0, τ_m^1	0.001, 0.1 ms	σ_p^1, σ_p^2	-10, 15 mV
$\boldsymbol{\theta_m}$	-38 mV	τ_n^0, τ_n^1	0, 2.4 ms	σ_q^1, σ_q^2	-15, 16 mV
$\boldsymbol{\theta_n}$	-42 mV	τ_p^0, τ_p^1	5, 0.33 ms	$I_{app_{GPe}}$	-0.009 nA
$\boldsymbol{\theta_p}$	-56 mV	τ_q^0, τ_q^1	0, 400 ms	$I_{app_{GPi}}$	0.006 nA

where *L* and *Diam* represent the length and diameter of the single compartment GP neurons. The calcium dynamics for the GP neurons are the model the same as for the STN neuron in equation A.5. GPe and GPi neurons are modelled using the same parameter values in Table 4 with the exception of their bias currents, $I_{app_{GPe}}$ and $I_{app_{GPi}}$.

1.3 Thalamic Neuron Model

The thalamic neuron model is from (Rubin and Terman, 2004) where the membrane potential of a thalamic neuron is described by

$$C_m \frac{dv_m}{dt} = -I_l - I_{Na} - I_K - I_T - \sum_k I_{syn}^k \qquad (A.8)$$

where C_m is the membrane capacitance, I_l is the leak current, I_{Na} is a sodium current, I_K is a potassium current, I_T is a low-threshold T-type calcium current and I_{syn} are synaptic currents. The equations governing the ionic currents are detailed in Table 5.

Table 5: Thalamic Neuron Model Equations

Current	Equation
$\boldsymbol{I_l}$	$g_l(v_m - E_l)$
$\boldsymbol{I_{Na}}$	$g_{Na} m_\infty^3 h(v_m - E_{Na})$
$\boldsymbol{I_K}$	$g_K\left(0.75 * (1-h)\right)^4 (v_m - E_K)$
$\boldsymbol{I_T}$	$g_T p_\infty^2 q(v_m - E_{Ca})$

The gating kinetics of the ionic conductances are governed by

$$\frac{dx}{dt} = \frac{x_\infty - x}{\tau_x} \qquad (A.9)$$

where x stands for h or q. The steady state activation and inactivation functions are given by

$$m_\infty = \frac{1}{1 + e^{-(v-37)/7}} \qquad (A.10)$$

$$h_\infty = \frac{1}{1 + e^{(v+41)/4}} \qquad (A.11)$$

$$p_\infty = \frac{1}{1 + e^{-(v+60)/6.2}} \qquad (A.12)$$

$$r_\infty = \frac{1}{1 + e^{(v+84)/4}} \qquad (A.13)$$

and the activation time constants are

$$\tau_h = \frac{1}{a_h + b_h} \qquad (A.14)$$

$$\tau_r = 28 + e^{-(v+25)/10.5} \qquad (A.15)$$

with specific functions a_h and b_h

$$a_h = 0.128 e^{-(v+46)/18} \qquad (A.16)$$

$$b_h = \frac{4}{1 + e^{-(v+23)/5}} \qquad (A.17)$$

Parameter values for the thalamic neuron model are summarized in Table 6:

Table 6: Thalamic Neuron Model Parameters

Parameter	Values
$\boldsymbol{C_m}$	100 µF/cm^2
$\boldsymbol{L}$	100 µm
$\boldsymbol{Diam}$	100 µm
$\boldsymbol{g_l}$	5 mS/cm^2
$\boldsymbol{g_{Na}}$	300 mS/cm^2
$\boldsymbol{g_K}$	500 mS/cm^2
$\boldsymbol{g_T}$	500 mS/cm^2
$\boldsymbol{E_l}$	-60 mV
$\boldsymbol{E_{Na}}$	50 mV
$\boldsymbol{E_K}$	-90 mV
$\boldsymbol{E_{Ca}}$	0 mV

where *L* and *Diam* represent the length and diameter of the single compartment thalamic neuron.

1.4 Cortical Pyramidal Neuron Model

The cortical pyramidal neuron model is modelled as a multicompartment neuron with the cortical soma compartment based on a model presented in (Pospischil et al., 2008) and the cortical axon compartments based on the model presented in (Foust et al., 2011). The membrane potential of each compartment in the cortical neuron model is described by the general equation

$$C_{m_x}\frac{dv_{m_x}}{dt} = -I_{l_x} - I_{Na_x} - I_{K_x} - I_{Kd_x} - I_{M_x} - \sum_k I_{syn_x}^k + I_{app_x} \qquad (A.18)$$

where x is the neuron compartment identifier, C_m is the membrane capacitance, I_l is the leak current, I_{Na} is a sodium current, I_K is a potassium current, I_{Kd} is a D-type potassium current, I_M is a slow, voltage-dependent potassium current, I_{syn} are synaptic currents and I_{app} is the neuron bias current. The equations governing the ionic currents are detailed in Tables 7 and 9.

1.4.1 Cortical Pyramidal Neuron Soma Model

The equations governing the ionic currents are detailed in Table 7 below.

Table 7: Cortical Pyramidal Neuron Soma Model Equations

Current	Equation
$\boldsymbol{I_{l_{soma}}}$	$g_{l_{soma}}(v_{m_{soma}} - E_{l_{soma}})$
$\boldsymbol{I_{Na_{soma}}}$	$g_{Na_{soma}}m^3h(v_{m_{soma}} - E_{Na_{soma}})$

$\boldsymbol{I_{K_{soma}}}$	$g_{K_{soma}} n^4 \left(v_{m_{soma}} - E_{K_{soma}}\right)$
$\boldsymbol{I_{M_{soma}}}$	$g_M p \left(v_m - E_{K_{soma}}\right)$

The gating kinetics of ionic conductances *m*, *h* and *n* are derived from a first order kinetic scheme and governed by

$$\frac{dx}{dt} = \alpha_x (1 - x) - \beta_x x \qquad (A.19)$$

where x stands for either *m*, *h* or *n*. The steady state activation functions and the time constants for *m*, *h* and *n* are given by

$$x_\infty = \frac{\alpha_x}{\alpha_x + \beta_x} \qquad (A.20)$$

$$\tau_x = \frac{1}{\alpha_x + \beta_x} \qquad (A.21)$$

where

$$\alpha_m = \frac{-0.32\left(v_{m_{soma}} - v_T - 13\right)}{e^{-\frac{v_{m_{soma}} - v_T - 13}{4}} - 1} \qquad (A.22)$$

$$\beta_m = \frac{0.28\left(v_{m_{soma}} - v_T - 40\right)}{e^{-\frac{v_{m_{soma}} - v_T - 40}{5}} - 1} \qquad (A.23)$$

$$\alpha_h = 0.128 e^{-\frac{v_{m_{soma}} - v_T - 17}{18}} \qquad (A.24)$$

$$\beta_h = \frac{4}{1 + e^{-\frac{v_{m_{soma}} - v_T - 40}{5}}} \qquad (A.25)$$

$$\alpha_n = \frac{-0.32\left(v_{m_{soma}} - v_T - 15\right)}{e^{-\frac{v_{m_{soma}} - v_T - 15}{5}} - 1} \qquad (A.26)$$

$$\beta_n = 0.5 e^{-\frac{v_{m_{soma}} - v_T - 10}{40}} \qquad (A.27)$$

The gating kinetics of the slow non-inactivating potassium current I_M is governed by

$$\frac{dp}{dt} = \frac{p_\infty - p}{\tau_p} \qquad (A.28)$$

and the steady state activation function and time constant is given by

$$p_\infty = \frac{1}{1 + e^{-\frac{(v_{m_{soma}}+35)}{10}}} \quad (A.29)$$

$$\tau_p = \frac{\tau_{max}}{3.3e^{\frac{v_{m_{soma}}+35}{20}} + e^{-\frac{v_{m_{soma}}+35}{20}}} \quad (A.30)$$

Parameter values for the cortical pyramidal neuron soma are summarized in Table 8:

Table 8: Cortical Pyramidal Neuron Soma Model Parameters

Parameter	Values
$\boldsymbol{C_{m_{soma}}}$	1 µF/cm^2
$\boldsymbol{L_{soma}}$	35 µm
$\boldsymbol{Diam_{soma}}$	25 µm
$\boldsymbol{g_{l_{soma}}}$	0.1 mS/cm^2
$\boldsymbol{g_{Na_{soma}}}$	50 mS/cm^2
$\boldsymbol{g_{K_{soma}}}$	5 mS/cm^2
$\boldsymbol{g_{M_{soma}}}$	0.07 mS/cm^2
$\boldsymbol{E_l}$	-70 mV
$\boldsymbol{E_{Na}}$	50 mV
$\boldsymbol{E_K}$	-100 mV
$\boldsymbol{v_T}$	-55 mV
$\boldsymbol{\tau_{max}}$	1000 ms
$\boldsymbol{I_{app_{soma}}}$	0.245 nA

where L_{soma} and $Diam_{soma}$ represent the length and diameter of the soma compartment and the soma bias current, $\boldsymbol{I_{app_{soma}}}$, value sets the cortical neuron firing rate within the beta-frequency band.

1.4.2 Cortical Pyramidal Neuron Axon Model

The equations governing the ionic currents for the cortical axon initial segment (AIS), axon myelinated segments, nodes of Ranvier segments and collateral segments are detailed in Table 9 below.

Table 9: Cortical Pyramidal Neuron Axon Model Equations

Current	Equation
$\boldsymbol{I_{l_{seg_{id}}}}$	$g_{l_{seg_{id}}}\left(v_{m_{seg_{id}}} - E_l\right)$
$\boldsymbol{I_{Na_{seg_{id}}}}$	$g_{Na_{seg_{id}}} m^3 h \left(v_{m_{seg_{id}}} - E_{Na}\right)$

$\boldsymbol{I_{K_{seg_{id}}}}$	$g_{K_{seg_{id}}} n \left(v_{m_{seg_{id}}} - E_K\right)$
$\boldsymbol{I_{Kd_{seg_{id}}}}$	$g_{Kd_{seg_{id}}} pq \left(v_{m_{seg_{id}}} - E_K\right)$

where seg_{id} specifies the axon compartment type. The gating kinetics of ionic conductances *m, h, n, p and q* are governed by

$$\frac{dx}{dt} = \frac{x_\infty - x}{\tau_x} \qquad (A.31)$$

where the steady state activation functions and time constants are described by

$$\tau_m = \frac{1}{\alpha_m + \beta_m}, \qquad m_\infty = \frac{\alpha_m}{\alpha_m + \beta_m} \qquad (A.32)$$

$$\tau_h = \frac{1}{\alpha_h + \beta_h}, \qquad h_\infty = \frac{1}{1 + e^{(v_{m_{seg_{id}}} + 60)/6.2}} \qquad (A.33)$$

$$\tau_n = \frac{1}{\alpha_n + \beta_n}, \qquad n_\infty = \frac{\alpha_n}{\alpha_n + \beta_n} \qquad (A.34)$$

$$\tau_p = 1\, ms, \qquad p_\infty = 1 - \frac{1}{1 + e^{(v_{m_{seg_{id}}} - V_{1/2}^{p})/q_p}} \qquad (A.35)$$

$$\tau_r = 1500\, ms, \qquad r_\infty = 1 - \frac{1}{1 + e^{(v_{m_{seg_{id}}} - V_{1/2}^{r})/q_r}} \qquad (A.36)$$

$$\alpha_m = \phi \frac{0.182\left(v_{m_{seg_{id}}} + 30\right)}{1 - e^{-(v_{m_{seg_{id}}} + 30)/8}}, \qquad \beta_m = -\phi \frac{0.124\left(v_{m_{seg_{id}}} + 30\right)}{1 - e^{(v_{m_{seg_{id}}} + 30)/8}} \qquad (A.37)$$

$$\alpha_h = \phi \frac{0.028\left(v_{m_{seg_{id}}} + 45\right)}{1 - e^{-(v_{m_{seg_{id}}} + 45)/6}}, \qquad \beta_h = -\phi \frac{0.0091\left(v_{m_{seg_{id}}} + 70\right)}{1 - e^{(v_{m_{seg_{id}}} + 70)/6}} \qquad (A.38)$$

$$\alpha_n = \phi \frac{0.01\left(v_{m_{seg_{id}}} - 30\right)}{1 - e^{-(v_{m_{seg_{id}}} - 30)/9}}, \qquad \beta_n = -\phi \frac{0.002\left(v_{m_{seg_{id}}} - 30\right)}{1 - e^{(v_{m_{seg_{id}}} - 30)/9}} \qquad (A.39)$$

$$\phi = Q_{10}^{T-23/10} \qquad (A.40)$$

where the Q_{10} effect, captured by ϕ, regulates temperature dependence, with Q_{10} = 2.3. Parameter values for the cortical axon model compartments are summarized in Table 10.

Table 10: Cortical Pyramidal Neuron Axon Model Parameters

Parameter	Values	Parameter	Values	Parameter	Values
$C_{m_{AIS}}$	0.8 µF/cm^2	$g_{l_{AIS}}$	0.033 mS/cm^2	$g_{Kd_{AIS}}$	15 mS/cm^2
$C_{m_{Myelin}}$	0.04 µF/cm^2	$g_{l_{Myelin}}$	0 mS/cm^2	$g_{Kd_{Myelin}}$	0 mS/cm^2
$C_{m_{Node}}$	0.8 µF/cm^2	$g_{l_{Node}}$	20 mS/cm^2	$g_{Kd_{Node}}$	7.2 mS/cm^2
$C_{m_{Collateral}}$	0.8 µF/cm^2	$g_{l_{Collateral}}$	0.033 mS/cm^2	$g_{Kd_{Collateral}}$	0.6 mS/cm^2
L_{AIS}	20 µm	$g_{Na_{AIS}}$	400 mS/cm^2	E_l	-70 mV
L_{Myelin}	500 µm	$g_{Na_{Myelin}}$	1 mS/cm^2	E_{Na}	60 mV
L_{Node}	2 µm	$g_{Na_{Node}}$	280 mS/cm^2	E_K	-90 mV
$L_{Collateral}$	500 µm	$g_{Na_{Collateral}}$	133.33 mS/cm^2	$V_{1/2}^{p}$	-43 mV
$Diam_{AIS}$	1.2 µm	$g_{K_{AIS}}$	2 mS/cm^2	$V_{1/2}^{r}$	-67 mV
$Diam_{Myelin}$	1.4 µm	$g_{K_{Myelin}}$	0 mS/cm^2	q_p	8
$Diam_{Node}$	1.2 µm	$g_{K_{Node}}$	0.5 mS/cm^2	q_r	7.3
$Diam_{Collateral}$	0.5 µm	$g_{K_{Collateral}}$	1 mS/cm^2		

1.5 Cortical Interneuron Model

The cortical interneuron model used was the single-compartment model from (Pospischil et al., 2008). The membrane potential of the cortical interneuron model is described by

$$C_m \frac{dv_m}{dt} = -I_l - I_{Na} - I_K - \sum_k I_{syn}^k + I_{app} \qquad (A.41)$$

where C_m is the membrane capacitance, I_l is the leak current, I_{Na} is a sodium current, I_K is a potassium current, I_{syn} are synaptic currents and I_{app} is the neuron bias current. The equations governing the ionic currents are detailed below in Tables 11.

Table 11: Cortical Interneuron Model Equations

Current	Equation
I_l	$g_l(v_m - E_l)$
I_{Na}	$g_{Na} m^3 h (v_m - E_{Na})$
I_K	$g_K n^4 (v_m - E_K)$

The gating kinetics of ionic conductances *m*, *h* and *n* are derived from a first order kinetic scheme and governed by

$$\frac{dx}{dt} = \alpha_x(1 - x) - \beta_x x \qquad (A.42)$$

where x stands for either *m*, *h* or *n*. The steady state activation functions and the time constants for *m*, *h* and *n* are given by

$$x_\infty = \frac{\alpha_x}{\alpha_x + \beta_x} \qquad (A.43)$$

$$\tau_x = \frac{1}{\alpha_x + \beta_x} \qquad (A.44)$$

where

$$\alpha_m = \frac{-0.32(v_m - v_T - 13)}{e^{-\frac{v_m - v_T - 13}{4}} - 1} \qquad (A.45)$$

$$\beta_m = \frac{0.28(v_m - v_T - 40)}{e^{-\frac{v_m - v_T - 40}{5}} - 1} \qquad (A.46)$$

$$\alpha_h = 0.128e^{-\frac{v_m - v_T - 17}{18}} \qquad (A.47)$$

$$\beta_h = \frac{4}{1 + e^{-\frac{v_m - v_T - 40}{5}}} \qquad (A.48)$$

$$\alpha_n = \frac{-0.32(v_m - v_T - 15)}{e^{-\frac{v_m - v_T - 15}{5}} - 1} \qquad (A.49)$$

$$\beta_n = 0.5e^{-\frac{v_m - v_T - 10}{40}} \qquad (A.50)$$

Parameter values for the cortical interneurons are summarized in Table 12:

Table 12: Cortical Interneuron Model Parameters

Parameter	Values
$\boldsymbol{C_m}$	1 μF/cm^2
$\boldsymbol{L}$	35 μm
$\boldsymbol{Diam}$	25 μm
$\boldsymbol{g_l}$	0.15 mS/cm^2
$\boldsymbol{g_{Na}}$	50 mS/cm^2
$\boldsymbol{g_K}$	10 mS/cm^2

$\boldsymbol{E_l}$	-70 mV
$\boldsymbol{E_{Na}}$	50 mV
$\boldsymbol{E_K}$	-100 mV
$\boldsymbol{v_T}$	-55 mV
$\boldsymbol{I_{app_{soma}}}$	0.07 nA

where *L* and *Diam* represent the length and diameter of the cortical interneuron.

1.6 Network Synaptic Connectivity

The details of the synaptic connections in the network are detailed in Table 13, where the number of connections between each presynaptic and postsynaptic population, the strength of the synaptic connections and the transmission delay are specified:

Table 13: Network Synaptic Connectivity Parameters

Pre	Post	# Connections	w_{syn} (μS)	t_{syn} (ms)
Cortical - Axon Node	Cortical - Interneuron	10	(0, 2.5e-3)	2
Cortical - Interneuron	Cortical - Soma	10	(0, 6.0e-3)	2
Cortical - Axon Collateral	STN Neuron	5	0.12	1
STN Neuron	GPe Neuron	1	0.11	4
GPe Neuron	GPe Neuron	1	0.015	4
GPe Neuron	STN Neuron	2	0.11	3
Striatal Neuron	GPe Neuron	1	0.01	1
STN Neuron	GPi Neuron	1	0.11	2
GPe Neuron	GPi Neuron	1	0.11	2
GPi Neuron	Thalamic Neuron	1	3	2
Thalamic Neuron	Cortical Neuron	1	5	2

where synaptic network connections followed a random connectivity pattern.
